# Multicenter Natural History Study and Long-Term Cochlear Implant Outcomes in Usher Syndrome Subtypes

**DOI:** 10.1097/AUD.0000000000001822

**Published:** 2026-04-23

**Authors:** Paul Emmerich Krumpoeck, Anselm Joseph Gadenstaetter, Natsumi Uehara, Takeshi Fujita, Nadia Giarbini, Maximilian Pawloff, Markus Ritter, Anna Landegger, Thomas Parzefall, Dominik Riss, Wolf-Dieter Baumgartner, Wolfgang Gstoettner, Christoph Arnoldner, Lukas David Landegger

**Affiliations:** 1 Christian Doppler Laboratory for Inner Ear Research, Department of Otolaryngology – Head and Neck Surgery, Vienna General Hospital, Medical University of Vienna, Vienna, Austria; 2 Department of Otolaryngology – Head and Neck Surgery, Vienna General Hospital, Medical University of Vienna, Vienna, Austria; 3 Department of Otolaryngology – Head and Neck Surgery, Kobe University Graduate School of Medicine, Kobe, Japan; 4 Audiology and Phoniatrics Center, hearLIFE Clinic Bolzano, Bolzano, Italy; 5 Department of Medical Sciences, Residency Program in Audiology and Phoniatrics, Postgraduate School of Medicine, University of Ferrara, Ferrara, Italy; 6 Department of Ophthalmology, Vienna General Hospital, Medical University of Vienna, Vienna, Austria; 7 Department of Otorhinolaryngology – Head and Neck Surgery, Medical University of Innsbruck, Innsbruck, Austria; 8 Department of Otolaryngology – Head and Neck Surgery, Stanford University School of Medicine, Palo Alto, California, USA.

**Keywords:** Audiology, Cochlear Implantation, Hearing, Natural history study, Pure-tone average, USH, Usher, Word recognition

## Abstract

**Objectives:**

Usher syndrome (USH) is an inherited disorder that causes bilateral sensorineural hearing loss (SNHL), retinitis pigmentosa, and vestibular defects. It represents the most common cause of combined deaf-blindness worldwide. Mutations in the nine known underlying genes are categorized into three types: USH type 1 has the earliest, most severe onset, while the much more common USH type 2 is comparatively mild with delayed onset and moderate symptoms. USH type 3 is very rare and exhibits variable clinical manifestations. The current standard of care for SNHL in USH is comprised of conventional hearing aids and/or cochlear implantation depending on the severity of symptoms. However, existing studies on treatment outcomes generally have rather short follow-up durations from a single institution and rarely distinguish between the individual USH subtypes.

**Design:**

This retrospective multicenter cohort study analyzed 655 audiograms of 33 patients (66 ears) with genetically confirmed USH. Patient-specific characteristics and treatments were correlated with pure-tone average thresholds and speech perception performance over a mean (SD) of 7 yrs and 10 mo (9 yrs and 11 mo) overall and 8 yrs and 2 mo (6 yrs and 3 mo) after cochlear implantation.

**Results:**

Conventional hearing aids improved thresholds on average (SD) by 20.1 (10.9) dB HL regardless of age, but aided hearing still deteriorated concurrently with the natural progression of SNHL. In patients with severe-to-profound SNHL or complete deafness, cochlear implantation rescued hearing to average thresholds of 37.9 (7.0) dB HL 1 yr post-implantation, 37.3 (10.3) dB HL after 2 years, and 27.5 (7.1) dB HL after 15 to 20 yrs. Every single patient who received an implant benefited from it, irrespective of the age at implantation or the causative USH mutation.

**Conclusions:**

With new gene therapies for USH in development, the findings reported here can serve as a benchmark for the comparison of novel treatments with the current clinical standard of care around the world. Larger-scale studies with more consistent monitoring of hearing and speech perception ability, as well as more extensive genetic testing, could further elucidate the benefits of current treatments for different (sub-)types of USH.

## INTRODUCTION

Usher syndrome (USH), a rare genetic disorder with an estimated global prevalence of 4 to 17/100,000 people, is the most common cause of deaf-blindness. It affects approximately 400,000 individuals worldwide and is responsible for 3 to 6% of all cases of hearing loss in children (Géléoc & El-Amraoui 2020; Delmaghani & El-Amraoui 2022). The condition follows an autosomal recessive pattern of inheritance and leads to sensorineural hearing loss (SNHL), varying degrees of visual impairment due to progressive retinitis pigmentosa (RP), and sometimes vestibular dysfunction (Castiglione & Möller 2022).

There are three main subgroups of USH (USH1-3), which are characterized by their clinical phenotype (Castiglione & Möller 2022). Patients with USH1, the most severe form, are born with profound bilateral SNHL, vestibular areflexia, and rapidly progressing RP that usually results in complete blindness before the age of 30 yrs (Nisenbaum et al. 2022).

USH2 is the most prevalent subgroup, comprising almost two-thirds of USH patients. Similar to USH1, the SNHL associated with USH2 is congenital and bilateral, albeit usually of moderate to severe degree. However, RP only begins to manifest itself in the second decade of life and vestibular pathologies are absent altogether (Nisenbaum et al. 2022; Colbert et al. 2024).

In contrast to the other two subgroups, patients with the rare USH3 (2–4% of cases), usually have a postlingual onset of variably severe progressive SNHL, develop RP in the first or second decade of life, and show vestibular symptoms in only half of all cases (Delmaghani & El-Amraoui 2022; Marouf et al. 2022). Aside from these three main subgroups, the recent discovery of a late-onset variant with a distinct genetic etiology has led to the recent establishment of the new USH4 (Velde et al. 2022).

As of today, the gold standard for diagnosis of USH is genetic testing and hitherto nine genes have been identified that are widely recognized to be specific for one of the clinical USH subtypes (Table 1). Certain mutations in these genes lead to degeneration of the sensory cells in the inner ear and retina, causing USH, while other mutations can also cause isolated forms of nonsyndromic deafness (Fuster-García et al. 2021; Castiglione & Möller 2022; Delmaghani & El-Amraoui 2022). Beyond these, there is a growing number of genes associated with pathologies that are similar to USH but do not fit the classical criteria (so-called “atypical USH”) (Delmaghani & El-Amraoui 2022; Walls et al. 2025).

**TABLE 1.** Definitive Usher syndrome subtypes

Usher Type	Auditory Phenotype	Vestibular Phenotype	Ocular Phenotype	Usher Subtype	Affected Gene	Protein	Function in Cochlea
Usher I	Profound SNHL	Complete vestibular areflexia	Rapidly progressing RP	USH1B	*MYO7A*	Myosin VIIa	Actin-based movement, stereocilia organization
				USH1C	*USH1C*	Harmonin	Hair cell bundle cohesion, protein scaffolding
				USH1D	*CDH23*	Cadherin 23	Tip-link component, mechanotransduction
				USH1F	*PCDH15*	Protocadherin 15	Tip-link component, mechanotransduction
				USH1G	*USH1G*	SANS	Tip-link complex assembly, scaffolding
Usher II	Moderate/severe SNHL	No vestibular defects	RP onset >10 yrs of age	USH2A	*USH2A*	Usherin	Basolateral membrane protein, linking of extracellular matrix to cytoskeleton, adhesion
				USH2C	*ADGRV1*	ADGRV1	Hair bundle development and maintenance, adhesion
				USH2D	*WHRN*	Whirlin	Stereocilia elongation, protein trafficking, scaffolding
Usher III	Variable degree of SNHL	Vestibular defects in ~50%	RP onset variable	USH3A	*CLRN1*	Clarin1	Hair cell synapse function, membrane trafficking

RP, retinitis pigmentosa; SNHL, sensorineural hearing loss.

To date, there is no curative therapy for the SNHL that occurs in USH. Current management relies on hearing aids in patients with sufficient residual hearing, but the only route to auditory rehabilitation for patients with severe-to-profound hearing loss is through cochlear implantation (Davies et al. 2021). Cochlear implants (CIs) directly stimulate the auditory nerve via an electrode array that is surgically implanted into the cochlea. This allows in turn to bypass the defective hair cells in USH. Especially patients with USH1 and USH2 can benefit from CIs with regard to auditory performance, oral communication skills, and cognitive ability (Jatana et al. 2013; Davies et al. 2021). However, in USH3 patients with severe hearing loss, CIs can also restore hearing thresholds from roughly 110 to ~35 dB HL (Pietola et al. 2012; Davies et al. 2021; Cornwall et al. 2024). Furthermore, it has been shown that bilateral cochlear implantation is superior to unilateral cochlear implantation (especially for directional hearing and speech comprehension in noise), and that interventions at a younger age (at least <3 yrs, ideally even younger) generally yield better outcomes. Therefore, the current standard of care in USH patients with congenital deafness is bilateral cochlear implantation at a prelingual age (Jatana et al. 2013; Davies et al. 2021; Remjasz-Jurek et al. 2023).

Due to the low prevalence of USH, studies of cochlear implantation in affected patients are often limited to small sample sizes. Also, for the majority of subjects in these studies, only individual institutions analyzed their respective cohorts, no genetic data was included, and therefore, no correlations between specific mutations, clinical progression, and postoperative CI outcomes could be established. However, this information would be essential not only for improving CI outcomes in the future, but also for providing a reference for comparison with possible future treatments (e.g., gene therapy) (Géléoc & El-Amraoui 2020; Davies et al. 2021).

For the present study, several patients with USH received CIs at some of the participating institutions over 30 yrs ago. In conjunction with genetic testing and long-term audiological monitoring of these individuals, this provides an ideal basis to investigate the natural progression of SNHL in USH and the efficacy of hearing aids and CIs for hearing rehabilitation.

## MATERIALS AND METHODS

We conducted a retrospective multicenter cohort study of 33 patients who presented at one of the three participating institutions (termed “study center A/B/C”) between January 1980 and August 2022 with USH verified through molecular genetic testing. Only patients with accessible results from at least one form of audiometric testing (see below) and genetically verified USH were included in this study.

### Data Collection

Demographic, disease-specific, and treatment-specific information was retrospectively gathered from patient records. This included date of birth, sex, and results obtained from diagnostic imaging (computed tomography, magnetic resonance imaging). Further, age at syndrome diagnosis, affected sides, USH subtype, mutated gene, total number of mutations, and zygosity were retrieved. Individual conservative and surgical treatment options were recorded with a special emphasis on cochlear implantation, for which data on age at surgery, side, electrode depth, postoperative complications, and implant and processor type were collected as well.

The audiometric results encompassed the age at testing, side, and air and bone conduction hearing thresholds. Four-frequency pure-tone averages (4-PTAs, from 0.5, 1, 2, and 4 kHz) were calculated from each of the air conduction measurements, as all patients suffered from pure SNHL and therefore no air-bone gaps were observed. Furthermore, speech perception percentages of numbers and word recognition scores (WRS) in the respective country standards were also recorded. Tests were done as free field measurements for young children and with headphones for older patients. They were further categorized into three groups according to their modality: unassisted, with traditional hearing aids, and with CIs.

### Statistical Analysis

Statistical analysis was performed using IBM SPSS Statistics 29.0 (IBM Corp., Armonk, NY, USA) and R version 4.3.1. The graphical illustrations were collated with GraphPad Prism version 10.4.1 (GraphPad Software Inc., Boston, MA, USA). Values are presented as individual numbers (n) with their respective percentages in brackets (%) for categorical variables or through either median with interquartile range (IQR) or mean with SD for numeric variables, depending on the normal distribution of the data determined via the Shapiro-Wilk test.

For testing the study’s hypotheses, we used the chi-squared test or a mixed-effects analysis for categorical variables. Hearing threshold (4-PTA) means were compared via two-tailed *t*-test or Wilcoxon matched-pairs signed rank test, depending on normality of the data according to the Shapiro-Wilk test. The log-rank (Mantel-Cox) test was used to compare CI-free time distributions between USH subtypes. The significance level for all tests was set a priori to *α* = 0.05. Correlations between two numeric variables (e.g., age and 4-PTA threshold) were modeled with simple linear regression and goodness of fit was determined with the coefficient of determination (*R*^2^). Corrections for multiple testing were done with Šídák’s multiple comparisons test.

## RESULTS

### Demographics of the Study Population

Overall, 33 patients with USH verified via molecular genetic testing were identified across our three study centers (25 from center A, 6 from center B, and 2 from center C). Seventeen (51.5%) patients were female and USH type 2 was the most frequently observed diagnosis (63.6%), with the most commonly affected gene being *USH2A* (60.6%). The majority of patients had 2 pathogenic mutations (28 of 33; 84.8%) and were heterozygous (29 of 33; 87.9%). A detailed overview of the individual mutations identified as well as their consequences can be found in Supplementary Table 1, Supplemental Digital Content, https://links.lww.com/EANDH/B888. Both ears from all 33 participants (66 ears in total) underwent further analysis regarding audiometric testing with and without hearing aids/CI. The overall frequencies of USH subtypes in our cohort and the treatments received are summarized in Table 2.

**TABLE 2.** USH subtypes and treatment frequencies in the present study’s population

Usher Type	Usher Subtype	Patients	Female sex	Ears	With Hearing Aid	With CI	Pre-CI Hearing Aid
Usher I	USH1B	6 (18.2%)	3 (50%)	12	4 (33.3%)	10 (83.3%)	4 (40%)
	USH1F	2 (6.1%)	0	4	4 (100%)	4 (100%)	4 (100%)
	USH1G	1 (3.0%)	1 (100%)	2	0	2 (100%)	0
Usher II	USH2A	20 (60.6%)	10 (50%)	40	37 (92.5%)	1 (2.5%)	0
	USH2C	1 (3.0%)	1 (100%)	2	0	1 (50%)	0
Usher III	USH3A	3 (9.1%)	2 (66.7%)	6	3 (50%)	3 (50%)	2 (66.7%)
		33 (100 %)	17 (51.5%)	66	48 (72.7%)	21 (31.8%)	10 (47.6%)

All numerical values are counts with their respective fractions in percent in brackets (%). Counts for hearing aid and CI treatment represent the entire patient history, so, for example, all four hearing aids of USH1B patients were subsequently replaced by CIs.

CI, cochlear implant; USH, Usher syndrome.

### Age and Audiometric Testing of the Study Population

**Fig. 1.**
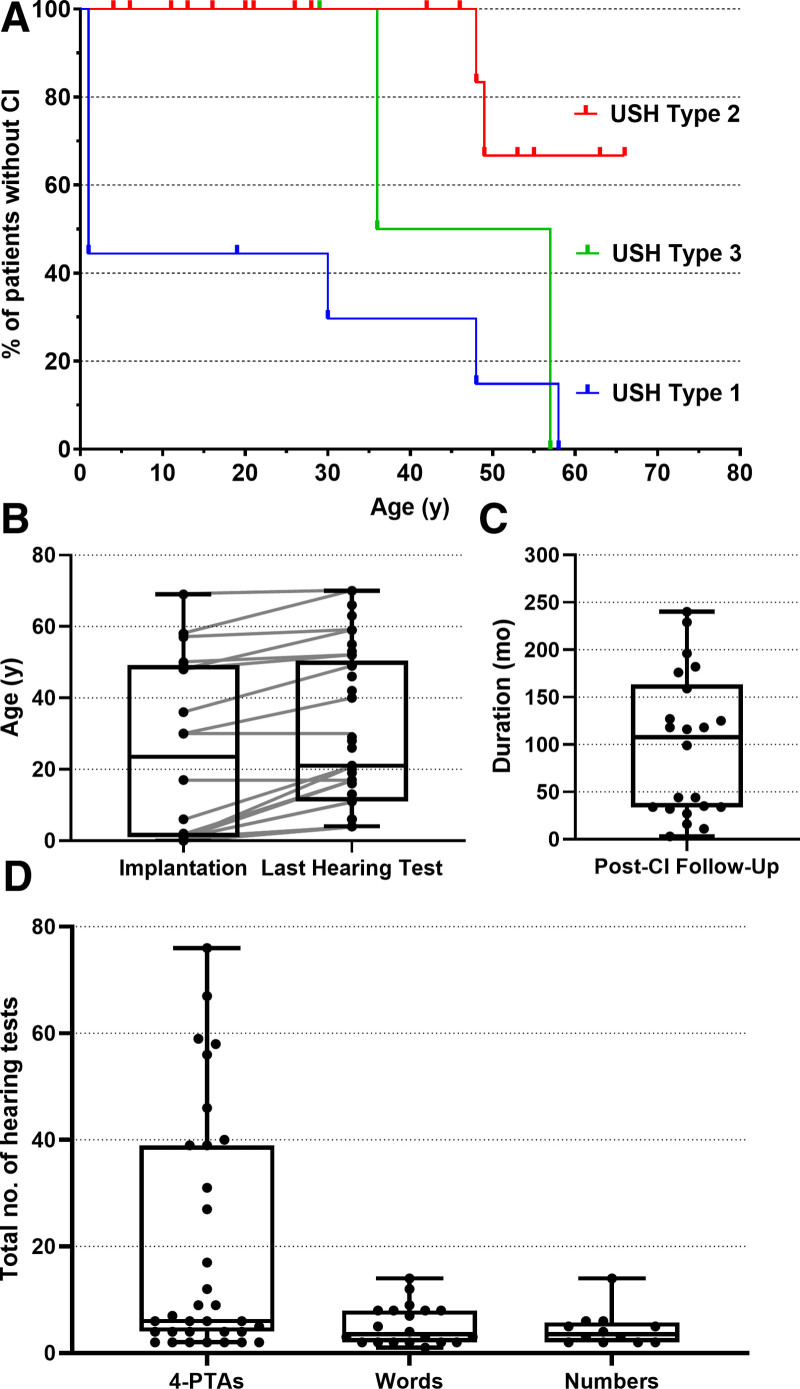
Age and audiometric testing of the study population. A, More than half of USH1 patients received a CI within the first 2 yrs of life, whereas more than two-thirds of USH2 patients remained unimplanted up to the end of their observation periods. Log-rank test of the CI-free time curves revealed significantly different distributions (*p* < 0.001). B, The median (IQR) age was 23.5 (1–49.25) yrs at cochlear implantation and 21 (11–50.5) yrs at the time of the latest audiometric testing (including all study patients). C, The median (IQR) post-CI follow-up duration was 107.5 (33.5–163.3) mo (mean [SD]: 98.4 [74.7]). D, The median number of 4-PTA measurements per patient (6 [4–39]) was much higher than the number of word recognition (3.5 [2–8]) and number recognition (3.5 [2–5.75]) measurements. All patients had 4-PTAs, while WRSs were performed at least once for 20 patients and number scores for 12 patients. Each data point per column represents an individual patient. 4-PTA indicates 4-frequency pure-tone average; CI, cochlear implant; IQR, interquartile range; USH, Usher syndrome; WRS, word recognition scores.

The median (IQR) ages at the last audiometric testing and at cochlear implantation varied widely; 21 (11 to 50.5) yrs at the last audiometric follow-up including all study patients and 23.5 (1 to 49.25) yrs at implantation (Fig. 1A and B). Of note, especially patients with USH type 1 received CIs at young ages. In fact, over half were implanted below 2 yrs of age (Fig. 1A). Conversely, many patients with USH types other than the above-mentioned USH1 received implants at higher ages—the oldest CI recipient was 69 yrs old at time of implantation—and several other individuals were not implanted at all. Also, for some patients, hearing (regardless of the treatment of choice) was regularly assessed for long periods of time into high ages. Consequently, the post-CI follow-up duration, which is the time from implantation to the latest audiometric testing, varies in a similar manner around a mean (SD) duration of 98.4 (74.7) mo (Fig. 1C). Overall, patients were followed for a median time of 48 (11 to 134.5) mo, or a mean (SD) of 94.2 (119) mo—more than 7 yrs—regardless of the treatment chosen. The minimum duration of observation was 0 days due to patients with only a single audiometry, while the longest duration was 546 mo (45.5 yrs). Among the audiometric measurements, pure-tone audiometries that allowed for 4-PTA calculations were performed most frequently, totaling 655 tests across the entire study population. Speech recognition tests were only done at study centers in countries where this was part of the clinical routine, totaling 105 tests across 20 patients for words and 54 across 12 patients for numbers (Fig. 1D). The mean number of 4-PTAs recorded per patient was 19.8 (22.8), followed by speech recognition tests with words (5.3 [3.7]) and with numbers (4.5 [3.4]).

### Course of Hearing Loss in USH With and Without Hearing Aids

**Fig. 2.**
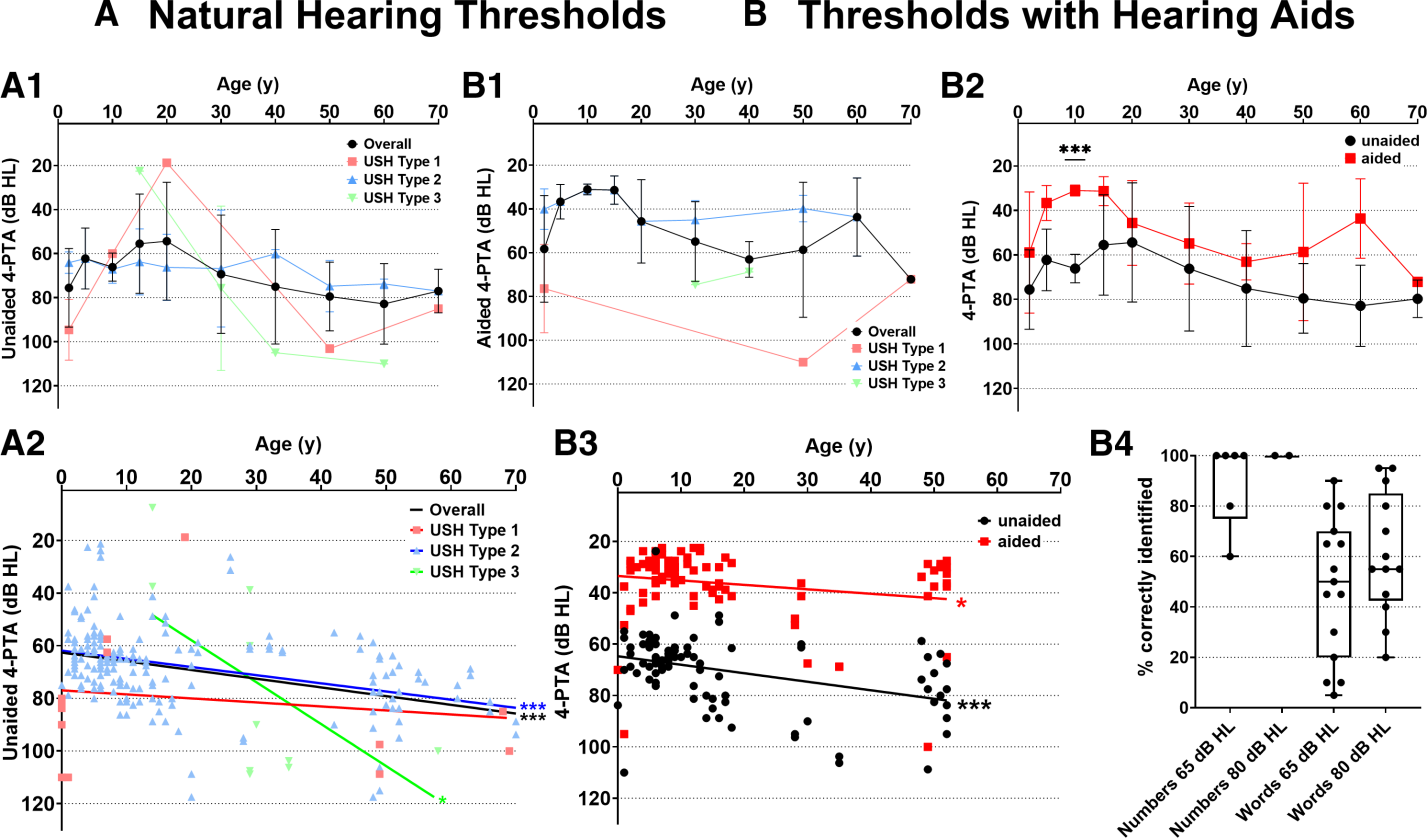
Pure-tone averages with and without conventional hearing aids and for different subtypes. A1, Unaided 4-PTA thresholds were best in 16- to 20-yr-old patients at 54.4 (23.1) dB HL and decrease with age; age groups: 0–2, 3–5, 6–10, 11–15, 16–20, 21–30, 31–40, 41–50, 51–60, and 61–70 yrs. A2, Unaided 4-PTA thresholds decrease with age in all USH subtypes (overall *p* < 0.001). B1, 4-PTA thresholds with hearing aids were best in 6- to 10-yr-old patients at 31.0 (2.1) dB HL. B2, 4-PTA thresholds with hearing aids were significantly better overall (two-tailed paired *t* test *p* < 0.001) and for 6- to 10-yr-old patients (mixed-effects analysis with Šídák’s multiple comparisons test *p* < 0.001). B3, 4-PTA thresholds with hearing aids decrease with age concurrently to unaided thresholds (aided *p* < 0.05, difference between slopes aided vs. unaided: *p* > 0.05). B4, Median aided WRS performance was 100% for numbers and 50% and 55% for monosyllabic words at intensities of 65 and 80 dB HL, respectively. 4-PTA, 4-frequency pure tone average; WRS, word recognition score. *p < 0.05, ***p < 0.001.

All USH-affected individuals suffered from SNHL, notably also those with USH type 3. The overall degree of hearing loss was moderately severe-to-profound, smallest at relatively young ages (3 to 20 yrs), and tended to worsen with increasing age (Fig. 2A1 and A2). Analysis of this trend via linear regression showed a significant decay of hearing overall (*p* < 0.001, *R*^2^ = 0.13) and in patients with USH Type 2 (*p* < 0.001, *R*^2^ = 0.15) and USH Type 3 (*p* = 0.035, *R*^2^ = 0.40), with similar but nonsignificant results for USH Type 1 (*p* > 0.05).

When supplied with a hearing aid, the patients’ hearing was improved by on average 20.1 (10.9) dB HL to mean (SD) thresholds of 31.0 (2.1) dB HL in patients 6 to 10 yrs of age (Fig. 2B1 and B2). This rescue was statistically significant when analyzing all tests together, regardless of the age at testing (two-tailed paired *t* test *p* < 0.001). The efficacy of hearing aids appeared to be greatest at young ages (6 to 10 yrs), where the differences between unaided and aided 4-PTA thresholds were highly significant (mixed-effects analysis with Šídák’s multiple comparisons test *p* < 0.001). The hearing aids remained effective throughout life, albeit the patients’ aided hearing also deteriorated after around 15 yrs of age in the course of the natural disease progression. This decay of aided hearing was also significant (*p* = 0.047, *R*^2^ = 0.04, see Fig. 2B3). Although word recognition for numbers was rarely assessed, these results were excellent with a median percentage of 100% correctly identified words. Scores for monosyllabic words were much lower at 50% for 65 dB HL and 55% for 80 dB HL (Fig. 2B4).

### Treatments in the Study Population

Of the 66 total ears, 48 (72.7%) received treatment with a conventional hearing aid (Table 2), and approximately half (n = 26; 54.2%) of these hearing aids were able to provide a level of hearing restoration reported as sufficient by the patient. For the other 22 ears (45.8%), there was either insufficient improvement or no further information reported, of which 10 (45.5%) were subsequently treated with cochlear implantation. In addition, 11 ears were supplied with a CI as first-line treatment for a grand total of 21 implanted ears (12 right ears, 9 left ears) in 12 patients within our study population (Table 2).

Before implantation, diagnostic imaging studies of the head were performed. At the time of this study, CT and magnetic resonance imaging scans were accessible for 10 and 9 patients, respectively, all of which revealed normal cochlear and retrocochlear conditions on both sides. The CIs received were all designed and manufactured by MED-EL (Innsbruck, Austria). The different models implanted were Synchrony 1 or 2 (45.5%), Sonata (27.3%), Pulsar (13.6%), Combi40+ (9.1%), and Concerto (4.5%). The processors used were from the Sonnet (78.6%) and Rondo (21.4%) series, with patients often switching to newer generations as they became available. Full insertion of the electrode array was achieved in all patients and proper placement was confirmed postoperatively via X-ray of the skull. During the postoperative stay at the hospital, there were no reports of complications in any case.

### Cochlear Implantation Rescues Hearing in All USH Subtypes

**Fig. 3.**
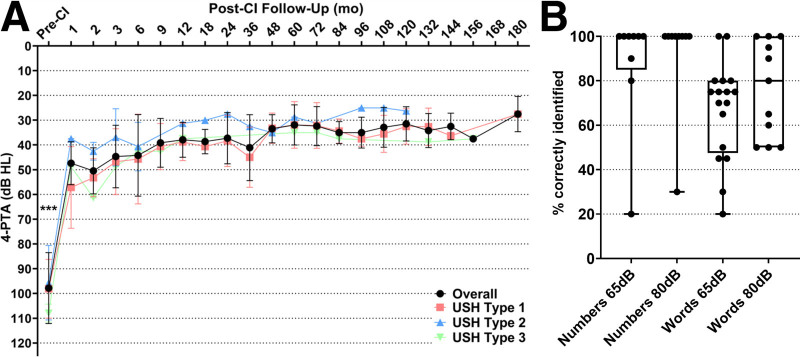
Cochlear implantation rescues hearing thresholds and speech perception in all USH subtypes. A, Mean 4-PTA thresholds are greatly reduced by cochlear implantation in all USH subtypes, from 97.8 (14.3) dB HL preoperatively to 47.4 (8.6) dB HL at 1 mo after CI surgery, 37.9 (7.0) dB HL after a year, and 37.3 (10.3) dB HL after 2 yrs (mixed-effects analysis with multiple comparisons: *p* < 0.001 for all timepoints compared with pre-CI, no significant differences between two adjacent post-CI timepoints); (B) In their latest speech recognition test, patients with CIs had median scores of 100% for numbers and 75% and 80% for monosyllabic words at intensities of 65 and 80 dB HL, respectively. 4-PTA indicates 4-frequency pure-tone average; CI, cochlear implant; USH, Usher syndrome. ***p < 0.001.

In accordance with the indication for CI surgery, all implanted patients had profound hearing loss with preoperative 4-PTA thresholds of 80 dB HL or more with a mean (SD) threshold of 97.8 (14.3) dB HL (Fig. 3A). The implant activation and first fitting took place on average 22.7 (9.1) days after surgery, at which point the mean hearing thresholds were already down to 47.4 (8.6) dB HL. All postoperative 4-PTA thresholds were measured from the implanted ear in isolation, with no hearing aid in the contralateral ear. In the following months, hearing improved slowly but steadily to thresholds of 37.9 (7.0) dB HL after a year, 37.3 (10.3) dB HL after 2 yrs, and reached 27.5 (7.1) dB HL at the longest observation period of 180 to 240 mo (15 to 20 yrs). The rescue and subsequent course of hearing thresholds was highly significant in the mixed-effects analysis with multiple comparisons, which yielded a *p* value <0.001 for all timepoints when compared with the preoperative state. Mixed-effects analysis also revealed no statistically significant difference in CI-aided hearing rescue between USH subtypes (*p* > 0.05 for all comparisons), and no outliers were observed across all the different mutations.

While no speech recognition tests could be performed preoperatively as the vast majority of patients suffered from nonfunctional hearing, they were conducted regularly after surgery. The latest results of each individual patient were plotted (Fig. 3B). The postoperative median percentage of correctly identified numbers was 100% at both the 65 and 80 dB HL intensities, respectively, whereas the median WRSs were 75% and 80%. This demonstrates that CIs were not only effective at restoring hearing in general but also greatly impacted speech perception.

### Cochlear Implantation Is Effective in Patients Where Hearing Aids Are Insufficient

**Fig. 4.**
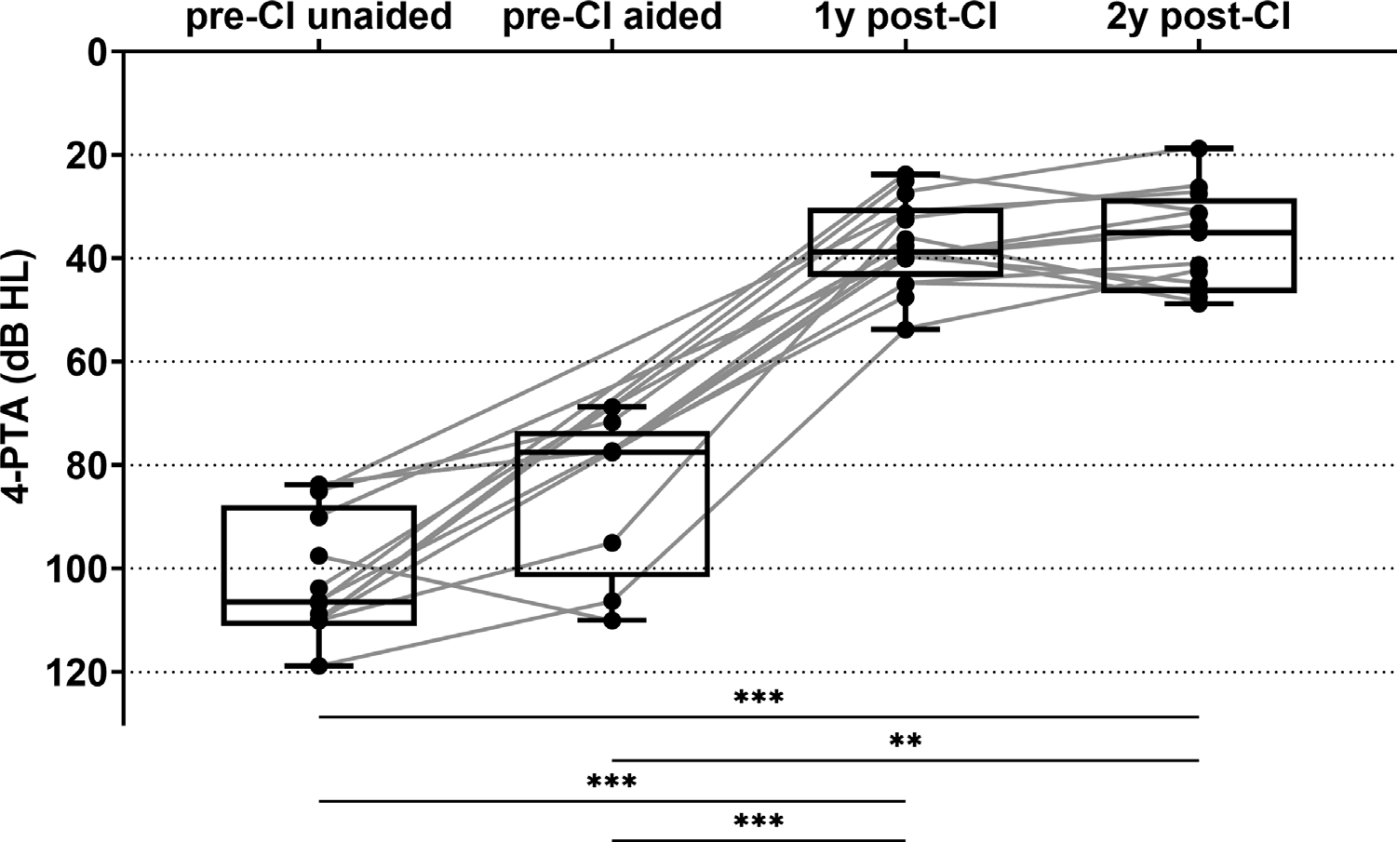
4-PTA thresholds are greatly improved in all CI recipients. Conventional hearing aids improved median (IQR) 4-PTA thresholds from 106.5 (88.75–110) dB HL to 77.5 (74.4–100.6) dB HL (IQR) (*p* > 0.05). CIs further improved hearing significantly compared with hearing aids, with thresholds at 38.75 (31.25–42.5) dB HL after 1 yr (*p* < 0.001) and 35 (29.4–45.6) dB HL (IQR) after 2 yrs (*p* = 0.004), as well as compared with the preoperative unaided state (*p* < 0.001 for both 1- and 2-yr periods). Statistical testing was done using mixed-effects analysis with Šídák’s multiple comparisons test. 4-PTA indicates 4-frequency pure tone average; CI, cochlear implant; IQR, interquartile range. **p < 0.01, ***p < 0.001.

Next, we compared the performance of CI patients with their preoperative performance, either with or without a hearing aid. Although hearing aids provided a nonsignificant mean (SD) improvement of 10.9 (5.3) dB HL in these patients (mixed-effects analysis with Šídák’s multiple comparisons test, *p* > 0.05), their median hearing thresholds of 77.5 (74.4 to 100.6) dB HL were still very high (Fig. 4). Particularly audiograms obtained decades ago at the participating centers did not consistently assess speech recognition, although the latter (in best-aided condition) represents the main indication for CI reimbursement in most countries these days.

Every single patient who received a CI benefited from it, regardless of whether there had been previous treatment with a hearing aid or not. The median (IQR) 4-PTA thresholds of CI-users at 1 yr after implantation were 38.75 (31.25 to 42.5) dB HL, which is significantly better than their preoperative hearing levels with (*p* < 0.001) or without hearing aid (*p* < 0.001, both *p* values obtained from mixed-effects analysis with Šídák’s multiple comparisons test). Their thresholds further improved to 35 (29.4 to 45.6) dB HL after 2 yrs with the implant.

### Cochlear Implantation Outcome Does Not Depend on Age at Implantation in Our Cohort

**Fig. 5.**
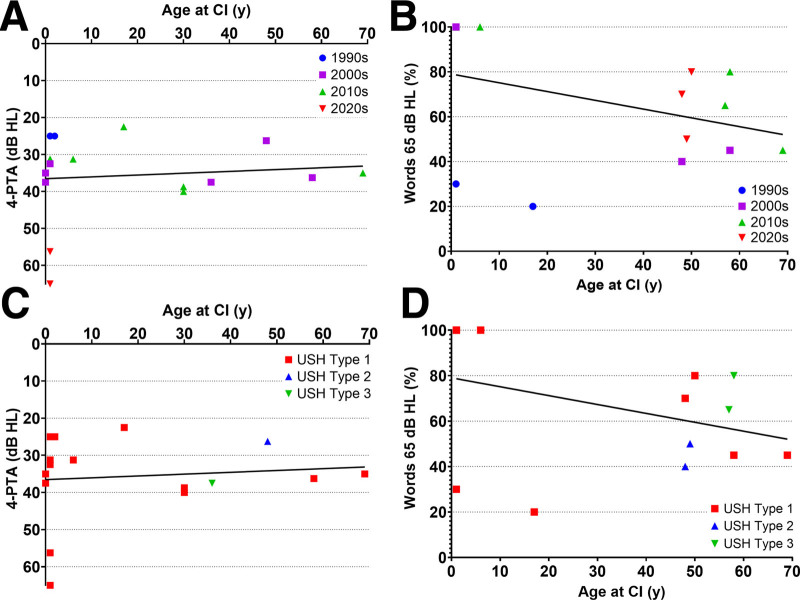
4-PTA threshold and WRS at the latest post-CI follow-up do not depend on age at implantation. A, Latest 4-PTA threshold for different ages at cochlear implantation stratified by decade of implantation. B, Latest WRS at 65 dB HL for different ages at cochlear implantation stratified by decade of implantation. C, Latest 4-PTA threshold for different ages at cochlear implantation stratified by USH subtype. D, Latest WRS at 65dB HL for different ages at cochlear implantation stratified by USH subtype. No significant correlation between age at implantation and either 4-PTA threshold or WRS at 65 dB HL was found (*p* > 0.05 for all comparisons). 4-PTA indicates 4-frequency pure-tone average; CI, cochlear implant; USH, Usher syndrome; WRS, Word recognition score.

Finally, we analyzed whether the age at implantation or the stage of CI development influenced the performance of the CIs. To investigate the latter, we stratified the data by the decade in which the surgery was performed (e.g., 1990s, 2000s, etc.) as a surrogate for the level of implant technology available at the time. For age, linear regression with the latest recorded 4-PTA threshold found no dependency (Fig. 5A), while regression with the latest WRS at 65 dB HL showed a slight decrease (Fig. 5B), albeit neither correlation was statistically significant (*p* > 0.05). There were even two patients with USH type 1 and no documented hearing aid use, one of whom was bilaterally implanted at 30 yrs of age and the other received sequential CIs at 58 and 69 yrs of age. In spite of the late implantations, both patients were able to achieve 4-PTA thresholds of 38.75/40.0 dB HL and 36.25/35.0 dB HL at their latest follow-ups, respectively. Similarly, no differences in the latest 4-PTA (Fig. 5A) or WRS (Fig. 5B) were observed between patients who received their CIs in different decades (*p* > 0.05 for all comparisons). To account for potential subtype-specific differences, a second analysis with separate stratification by USH subtype was conducted, yielding similar results (Fig. 5C and D, *p* > 0.05 for all comparisons). In addition, these analyses were restricted to patients with at least 12 mo of post-implantation data to eliminate any potential bias introduced by very brief follow-up durations.

## DISCUSSION

USH is a very heterogeneous condition, both genetically and clinically. Ocular and vestibular symptoms vary between subtypes and different mutations of the various underlying genes (Castiglione & Möller 2022; Nisenbaum et al. 2022). The same is true for the SNHL, as could also be observed in the present study. The few hearing tests of unimplanted patients with USH type 1 show an above-average degree of SNHL already at a very young age both with and without hearing aids (Fig. 2A1, A2, and B1), although the fact that only so few tests are available—because 16 of 18 ears (88.9%) received a CI—speaks for itself. Multiple implantations of USH1 patients were performed at very young ages in the past few years, which also explains the great variabilities of the age at implantation and the post-CI follow-up duration in our cohort. The CI performance in our study was favorable across all subtypes (Fig. 3), which is similar to previous studies (Davies et al. 2021; Remjasz-Jurek et al. 2023; Cornwall et al. 2024; Fehrmann et al. 2024). Conversely, patients with USH type 2 had less severe hearing loss as observed in previous studies. In fact, nearly all patients with this USH subtype could be sufficiently treated with a hearing aid (88.1% of ears) and only 2 ears were supplied with a CI. One of the patients also requested CIs due to poor performance of his hearing aids on both ears, which had not yet been implanted at the time of writing this article. This means that for the greater part of our USH type 2 patients, the hearing loss observed was mild enough to be responsive to conventional hearing aid treatment. Our findings complement those of Busi & Castiglione (2024), where only 10 of 32 CI recipients (31.25%) had USH type 2A, although this subtype accounts for about two-thirds of all cases (Nisenbaum et al. 2022; Busi & Castiglione 2024). Moreover, USH2A patients in that study also had by far the best post-implantation hearing in their analysis with an odds ratio for an outcome rated as “excellent” of 8.07 (*p* < 0.01) and the two aforementioned ears in our cohort had the best 4-PTA thresholds with CI of all ears over the entire postoperative follow-up period (Fig. 3A).

As USH type 3 is the rarest subtype of USH, there were only 3 patients with this genotype in our study population and therefore only a limited number of audiometric tests were available. However, the results were similarly variable as in previous studies with unaided thresholds that were as good as 22.5 dB HL to ones as bad as 110.0 dB HL (Marouf et al. 2022; Nisenbaum et al. 2022). The reason for this variability is currently unknown, although it has been suggested that alternative splicing or modifier genes may play a role (Pietola et al. 2012). Cochlear implantation proved just as effective as for the other two subtypes (Fig. 3A), which is in line with prior investigations that found postoperative auditory thresholds of 34 dB HL and speech recognition scores between 52% and 75% (Loundon et al. 2003; Pietola et al. 2012; Marouf et al. 2022). Overall, the benefits of cochlear implantation in our study were comparable to those reported in the current literature, although our long follow-up durations indicate that these benefits are in fact stable over many years (Jatana et al. 2013; Hoshino et al. 2017; Davies et al. 2021; Remjasz-Jurek et al. 2023; Cornwall et al. 2024; Fehrmann et al. 2024). These outcomes are generally favorable, but may not be quite as good as in recipients with nonsyndromic etiologies of SNHL. However, currently published results are somewhat conflicting regarding CI performance in USH versus other syndromic or nonsyndromic etiologies (e.g., *GJB2* mutations), and a general consensus is yet to be established, with recent analyses suggesting that subject-specific clinical factors may outweigh genetic differences (Sahoo et al. 2022; Caragli et al. 2023; Remjasz-Jurek et al. 2023; Tropitzsch et al. 2023; Wu 2024; Fehrmann et al. 2025).

Fortunately, not all patients with USH require a CI immediately or even at any point in life. As mentioned, especially patients with subtypes USH2 and USH3 often retain enough residual hearing ability to be treatable with conventional hearing aids. While hearing aids only amplify the incoming sound and do not restore hearing ability itself, they can still improve sound perception and communication in many patients, either permanently or at least temporarily before the loss of cochlear hair cells eventually progresses to necessitate a CI. In our study cohort, auditory thresholds were reduced by an average of 20.1 (10.9) dB HL across all ages and subtypes, although patients under ~30 yrs benefitted most from hearing aids (Fig. 2A2 and B3). At higher ages, aided hearing started to deteriorate concurrently to unaided hearing, and, likely due to follow-up bias, the number of audiometries available plummeted, making it difficult to draw conclusions. There was also no information on the specific types of hearing aids (e.g., analog versus digital processors, in-the-ear versus behind-the-ear) or the age when patients were first provided with the devices and the subsequent duration of use in these patients, which are important factors that further determine their utility. Nevertheless, the age-related dynamic makes sense mechanistically for similar reasons as in cochlear implantation (see next paragraph) and has also previously been observed specifically for hearing aids (Ching et al. 2018).

Unexpectedly, in our population, we did not identify the well-established significant dependence of the performance with CI on the age at implantation (Jatana et al. 2013; Hoshino et al. 2017; Davies et al. 2021; Remjasz-Jurek et al. 2023; Fehrmann et al. 2024), or the stage of CI development. Although the worst two performances in terms of WRSs were achieved after cochlear implantation in the 1990s, the overall outcomes were not significantly different from those achieved with more modern devices in the 2000s, 2010s, or 2020s. This suggests that while CI technology has undoubtedly advanced, even older-generation devices provided substantial and lasting benefits to patients with USH. Furthermore, it indicates that the lack of an age effect was not confounded by patients in these various stages of life having received CIs from different technological eras. Even individuals who were implanted at ages up to 69 yrs were able to achieve hearing similar to patients implanted during early childhood. However, in contrast to our study, the previous literature that established this age-dependence had much lower average ages at implantation or were entirely conducted in pediatric populations (Jatana et al. 2013; Davies et al. 2021; Remjasz-Jurek et al. 2023; Fehrmann et al. 2024). As a result, there are currently very few data for USH patients implanted during adulthood or even later. A retrospective study of outcomes of late implantation conducted by Hoshino et al. (2017) reported average hearing thresholds of 35 dB HL at 1 yr post-implantation, which is similar to both our results and the previous research with children (Hoshino et al. 2017). Their average age at implantation was 18.9 yrs, which is still relatively young compared with many of the patients in our cohort. More data would be required to achieve further insights into the influence of age and duration of deafness on the benefits of cochlear implantation in older individuals.

Although our data included many audiometric tests and detailed insights about patients with different mutations, of various age groups, and from different countries, there are still several shortcomings that limit their representativeness and warrant further investigation going forward. For one, the speech recognition tests were only performed by some of the study centers and were often done sporadically in many patients. They were sometimes omitted by audiometrists when the patients’ 4-PTA results were either similar to prior audiograms or if hearing loss was profound so that nearly no word recognition was likely (e.g., >100 dB HL). The words and numbers were also not just presented at the commonly chosen intensities of 65 and 80 dB HL but at many different levels from 15 dB HL all the way up to 110 dB HL. This made it difficult to “standardize” the results, which is why we chose to only include speech tests conducted at the two aforementioned intensities. While all of this is part of normal clinical practice, it also makes it difficult to pinpoint and follow the patients’ speech recognition abilities from the pre- to the post-implantation period and in the years thereafter.

Another unfortunate circumstance of retrospective clinical studies is that they rely on long-term close monitoring of participants for optimally representative results. This is especially relevant in our case, as some patients were indeed followed rigorously for long periods of time, but others only presented at our clinics recently or chose not to attend their scheduled follow-up appointments for many years. Therefore, our follow-up durations are long in some cases (up to 240 mo = 20 yrs), but extremely short in others at a minimum of 2 mo. The number of audiometries performed varies similarly among patients, resulting in varying accuracy of data representing individual patients.

When it comes to the diagnosis of USH, current comprehensive gene panels for hearing loss include all nine known USH genes. However, this testing is unfortunately not routinely performed for all patients with a clinical diagnosis (Colbert et al. 2024), which is especially true for diagnoses that were made decades ago. Therefore, there were many patients with clinical “diagnoses” of USH that had to be excluded from this study because no records of genetic testing were available. For others whose diagnoses were established via genetic testing at external institutes, there were records available, but these could not be accessed due to administrative, legal, or ethical reasons. This meant that many additional patients could not be included in the type-specific analyses and we identified no patients with subtypes USH1C, USH1D, or USH2D. Moreover, the sample sizes for some of the other subtypes were also rather small, which all results in our overview not being as comprehensive as might be possible with prospective trials. In the future, standardized gene panels will hopefully be expanded and see more frequent use in clinical practice with routine reimbursement by insurances as already standard in several countries. This will make it easier to assess the frequency of specific mutations and to correlate the mutations and subtypes to clinical outcomes.

Increased frequency and broad implementation of genetic testing are not just the way to faster and more accurate diagnoses, but may also allow us to understand the underlying mutations better, which is critical for the development of new causal treatments (Colbert et al. 2024). In fact, gene therapy approaches can open up new possibilities directly restoring the function of defective genes and are already being investigated for many forms of USH (Jiang et al. 2023). Among the various viral and nonviral vectors available, the overwhelming majority of studies for the treatment of SNHL in USH so far have employed adeno-associated viral vectors (Jiang et al. 2023).

In mouse models, adeno-associated viral vectors have successfully replaced missing genes in inner ear cells and thereby restored hearing to auditory brainstem response thresholds between 90 and 25 dB for the genes *MYO7A* (Lau et al. 2023; Schott et al. 2023), *USH1C* (Pan et al. 2017), *PCDH15* (Ivanchenko et al. 2023, 2024), *USH1G* (Emptoz et al. 2017; Lahlou et al. 2024), *WHRN* (Chien et al. 2016; Isgrig et al. 2017), and *CLRN1* (Geng et al. 2017; Dulon et al. 2018; György et al. 2019; Ivanchenko et al. 2021). Other approaches have successfully corrected mutations on a molecular level, which has been done for *USH1C* (Lentz et al. 2020), *PCDH15* (Liu et al. 2022; Peters et al. 2023), and *MYO7A* (Pan et al. 2023). Moreover, recent first trials in non-human primates have yielded promising results with regard to transduction of the inner ear and retina, encouraging the translation of these new therapeutics into first clinical trials (György et al. 2019; Ivanchenko et al. 2021, 2024). Nevertheless, gene therapies still face several challenges like a lack of representative animal models for several USH subtypes, limited patient populations and clinical endpoints for future clinical trials, and economic and regulatory hurdles, before they can become broadly available in clinical practice (Bhat et al. 2024).

To conclude, our study observed hearing loss in individuals with USH that was variable in its severity across different underlying mutations and progressed with age. Treatment with conventional hearing aids was a viable tool that improved hearing by an average of 20.1 (10.9) dB HL, but aided hearing deteriorated similarly to unaided hearing with increasing age. For patients who had profound hearing loss and those who did not sufficiently benefit from hearing aids, cochlear implantation provided a suitable alternative, as every single patient who received a CI benefited from it regardless of age, USH subtype or mutation, or prior hearing aid usage. These clinical outcomes are valuable for counseling patients about the optimal choice of treatment for their specific case and to provide them with realistic prognoses. Furthermore, with the rise of new gene therapies for USH, this study could serve as a benchmark for the comparison of future clinical trials with the current standard of care.

## ACKNOWLEDGMENTS

The financial support by the Austrian Federal Ministry of Labour and Economy, the National Foundation for Research, Technology and Development, and the Christian Doppler Research Association is gratefully acknowledged.

## Supplementary Material

## References

[R1] Bhat R. Nallamothu B. Shethia F. Chhaya V. Khambholja K. (2024). Key challenges in developing a gene therapy for Usher syndrome: Machine-assisted scoping review. J Community Genet, 15, 735–747. 39549230 10.1007/s12687-024-00749-0 PMC11645336

[R2] Busi M., & Castiglione A. (2024). Navigating the usher syndrome genetic landscape: An evaluation of the associations between specific genes and quality categories of cochlear implant outcomes. Audiol Res, 14, 254–263. 38525684 10.3390/audiolres14020023 PMC10961690

[R3] Caragli V. Monzani D. Genovese E. Palma S. Persico A. M. (2023). Cochlear implantation in children with additional disabilities: A systematic review. Children (Basel) 10, 1653. 37892316 10.3390/children10101653 PMC10605071

[R4] Castiglione A., & Möller C. (2022). Usher syndrome. Audiol Res, 12, 42–65. 35076463 10.3390/audiolres12010005 PMC8788290

[R5] Chien W. W. Isgrig K. Roy S. Belyantseva I. A. Drummond M. C. May L. A. Fitzgerald T. S. Friedman T. B. Cunningham L. L. (2016). Gene therapy restores hair cell stereocilia morphology in inner ears of deaf whirler mice. Mol Ther, 24, 17–25. 26307667 10.1038/mt.2015.150 PMC4754541

[R6] Ching T. Y. C. Dillon H. Leigh G. Cupples L. (2018). Learning from the longitudinal outcomes of children with hearing impairment (LOCHI) study: Summary of 5-year findings and implications. Int J Audiol, 57(sup2, S105–S111. 10.1080/14992027.2017.1385865 PMC5897193 29020839

[R7] Colbert B. M. Smeal M. Cromar Z. J. Rosa P. Blanton S. H. Lam B. L. Liu X. Z. (2024). Prevalence of molecular diagnoses for Usher syndrome and the need for coordinated care. Laryngoscope, 135, 1777–1780. 39560289 10.1002/lary.31911 PMC11980960

[R8] Cornwall H. L. Lam C. M. Chaudhry D. Muzaffar J. Monksfield P. Bance M. L. (2024). Outcomes of cochlear implantation in Usher syndrome: A systematic review. Eur Arch Otorhinolaryngol, 281, 1115–1129. 37930386 10.1007/s00405-023-08304-2 PMC10858075

[R9] Davies C. Bergman J. Misztal C. Ramchandran R. Mittal J. Bulut E. Shah V. Mittal R. Eshraghi A. A. (2021). The outcomes of cochlear implantation in Usher syndrome: A systematic review. J Clin Med, 10, 2915. 34209904 10.3390/jcm10132915 PMC8267700

[R10] Delmaghani S., & El-Amraoui A. (2022). The genetic and phenotypic landscapes of Usher syndrome: From disease mechanisms to a new classification. Hum Genet, 141, 709–735. 35353227 10.1007/s00439-022-02448-7 PMC9034986

[R11] Dulon D. Papal S. Patni P. Cortese M. Vincent P. F. Tertrais M. Emptoz A. Tlili A. Bouleau Y. Michel V. Delmaghani S. Aghaie A. Pepermans E. Alegria-Prevot O. Akil O. Lustig L. Avan P. Safieddine S. Petit C. El-Amraoui A. (2018). Clarin-1 gene transfer rescues auditory synaptopathy in model of Usher syndrome. J Clin Invest, 128, 3382–3401. 29985171 10.1172/JCI94351 PMC6063508

[R12] Emptoz A. Michel V. Lelli A. Akil O. Boutet de Monvel J. Lahlou G. Meyer A. Dupont T. Nouaille S. Ey E. Franca de Barros F. Beraneck M. Dulon D. Hardelin J. P. Lustig L. Avan P. Petit C. Safieddine S. (2017). Local gene therapy durably restores vestibular function in a mouse model of Usher syndrome type 1G. Proc Natl Acad Sci USA, 114, 9695–9700. 28835534 10.1073/pnas.1708894114 PMC5594693

[R13] Fehrmann M. L. A. Haer-Wigman L. Kremer H. Yntema H. G. Thijssen M. E. G. Mylanus E. A. M. Huinck W. J. Lanting C. P. Pennings R. J. E. (2025). Cochlear implantation outcomes in genotyped subjects with sensorineural hearing loss. J Assoc Res Otolaryngol, 26, 331–348. 40268851 10.1007/s10162-025-00987-0 PMC12133674

[R14] Fehrmann M. L. A. Lanting C. P. Haer-Wigman L. Yntema H. G. Mylanus E. A. M. Huinck W. J. Pennings R. J. E. (2024). Long-term outcomes of cochlear implantation in Usher syndrome. Ear Hear, 45, 1542–1553. 38987893 10.1097/AUD.0000000000001544 PMC11487040

[R15] Fuster-García C. García-Bohórquez B. Rodríguez-Muñoz A. Aller E. Jaijo T. Millán J. M. García-García G. (2021). Usher syndrome: Genetics of a human ciliopathy. Int J Mol Sci, 22, 6723. 34201633 10.3390/ijms22136723 PMC8268283

[R16] Géléoc G. G. S., & El-Amraoui A. (2020). Disease mechanisms and gene therapy for Usher syndrome. Hear Res, 394, 107932. 32199721 10.1016/j.heares.2020.107932

[R17] Geng R. Omar A. Gopal S. R. Chen D. H. Stepanyan R. Basch M. L. Dinculescu A. Furness D. N. Saperstein D. Hauswirth W. Lustig L. R. Alagramam K. N. (2017). Modeling and preventing progressive hearing loss in Usher syndrome III. Sci Rep, 7, 13480. 29044151 10.1038/s41598-017-13620-9 PMC5647385

[R18] György B. Meijer E. J. Ivanchenko M. V. Tenneson K. Emond F. Hanlon K. S. Indzhykulian A. A. Volak A. Karavitaki K. D. Tamvakologos P. I. Vezina M. Berezovskii V. K. Born R. T. O’Brien M. Lafond J. F. Arsenijevic Y. Kenna M. A. Maguire C. A. Corey D. P. (2019). Gene transfer with AAV9-PHP.B rescues hearing in a mouse model of Usher syndrome 3A and transduces hair cells in a non-human primate. Mol Ther Methods Clin Dev, 13, 1–13. 30581889 10.1016/j.omtm.2018.11.003 PMC6297893

[R19] Hoshino A. C. Echegoyen A. Goffi-Gomez M. V. Tsuji R. K. Bento R. F. (2017). Outcomes of late implantation in usher syndrome patients. Int Arch Otorhinolaryngol, 21, 140–143. 28382120 10.1055/s-0036-1583306 PMC5375700

[R20] Isgrig K. Shteamer J. W. Belyantseva I. A. Drummond M. C. Fitzgerald T. S. Vijayakumar S. Jones S. M. Griffith A. J. Friedman T. B. Cunningham L. L. Chien W. W. (2017). Gene therapy restores balance and auditory functions in a mouse model of Usher syndrome. Mol Ther, 25, 780–791. 28254438 10.1016/j.ymthe.2017.01.007 PMC5363211

[R21] Ivanchenko M. V. Hanlon K. S. Hathaway D. M. Klein A. J. Peters C. W. Li Y. Tamvakologos P. I. Nammour J. Maguire C. A. Corey D. P. (2021). AAV-S: A versatile capsid variant for transduction of mouse and primate inner ear. Mol Ther Methods Clin Dev, 21, 382–398. 33869656 10.1016/j.omtm.2021.03.019 PMC8044388

[R22] Ivanchenko M. V. Hathaway D. M. Klein A. J. Pan B. Strelkova O. De-la-Torre P. Wu X. Peters C. W. Mulhall E. M. Booth K. T. Goldstein C. Brower J. Sotomayor M. Indzhykulian A. A. Corey D. P. (2023). Mini-PCDH15 gene therapy rescues hearing in a mouse model of Usher syndrome type 1F. Nat Commun, 14, 2400. 37100771 10.1038/s41467-023-38038-y PMC10133396

[R23] Ivanchenko M. V. Hathaway D. M. Mulhall E. M. Booth K. T. Wang M. Peters C. W. Klein A. J. Chen X. Li Y. György B. Corey D. P. (2024). PCDH15 dual-AAV gene therapy for deafness and blindness in Usher syndrome type 1F models. J Clin Investig, 134, e177700. 39441757 10.1172/JCI177700 PMC11601915

[R24] Jatana K. R. Thomas D. Weber L. Mets M. B. Silverman J. B. Young N. M. (2013). Usher syndrome. Otol Neurotol, 34, 484–489. 23442567 10.1097/MAO.0b013e3182877ef2

[R25] Jiang L. Wang D. He Y. Shu Y. (2023). Advances in gene therapy hold promise for treating hereditary hearing loss. Mol Ther, 31, 934–950. 36755494 10.1016/j.ymthe.2023.02.001 PMC10124073

[R26] Lahlou G. Calvet C. Simon F. Michel V. Alciato L. Plion B. Boutet de Monvel J. Lecomte M. J. Beraneck M. Petit C. Safieddine S. (2024). Extended time frame for restoring inner ear function through gene therapy in Usher1G preclinical model. JCI Insight, 9, 1–14. 10.1172/jci.insight.169504 PMC10967394 38194286

[R27] Lau S. C. Grati M. Isgrig K. Sinan M. Calabro K. R. Zhu J. Ishibashi Y. Ozgur Z. Wafa T. Belyantseva I. A. Fitzgerald T. Friedman T. B. Boye S. L. Boye S. E. Chien W. W. (2023). Dual-AAV vector-mediated expression of MYO7A improves vestibular function in a mouse model of Usher syndrome 1B. Mol Ther Methods Clin Dev, 30, 534–545. 37693946 10.1016/j.omtm.2023.08.012 PMC10491803

[R28] Lentz J. J. Pan B. Ponnath A. Tran C. M. Nist-Lund C. Galvin A. Goldberg H. Robillard K. N. Jodelka F. M. Farris H. E. Huang J. Chen T. Zhu H. Zhou W. Rigo F. Hastings M. L. Géléoc G. S. G. (2020). Direct delivery of antisense oligonucleotides to the middle and inner ear improves hearing and balance in usher mice. Mol Ther, 28, 2662–2676. 32818431 10.1016/j.ymthe.2020.08.002 PMC7704764

[R29] Liu L. Zou L. Li K. Hou H. Hu Q. Liu S. Li J. Song C. Chen J. Wang S. Wang Y. Li C. Du H. Li J. L. Chen F. Xu Z. Sun W. Sun Q. Xiong W. (2022). Template-independent genome editing in the Pcdh15av-3j mouse, a model of human DFNB23 nonsyndromic deafness. Cell Rep, 40, 111061. 35830793 10.1016/j.celrep.2022.111061

[R30] Loundon N. Marlin S. Busquet D. Denoyelle F. Roger G. Renaud F. Garabedian E. N. (2003). Usher syndrome and cochlear implantation. Otol Neurotol, 24, 216–221. 12621335 10.1097/00129492-200303000-00015

[R31] Marouf A. Johnson B. Alagramam K. N. (2022). Usher syndrome IIIA: A review of the disorder and preclinical research advances in therapeutic approaches. Hum Genet, 141, 759–783. 35320418 10.1007/s00439-022-02446-9

[R32] Nisenbaum E. Thielhelm T. P. Nourbakhsh A. Yan D. Blanton S. H. Shu Y. Koehler K. R. El-Amraoui A. Chen Z. Lam B. L. Liu X. (2022). Review of genotype-phenotype correlations in Usher syndrome. Ear Hear, 43, 1–8. 34039936 10.1097/AUD.0000000000001066 PMC8613301

[R33] Pan B. Askew C. Galvin A. Heman-Ackah S. Asai Y. Indzhykulian A. A. Jodelka F. M. Hastings M. L. Lentz J. J. Vandenberghe L. H. Holt J. R. Géléoc G. S. (2017). Gene therapy restores auditory and vestibular function in a mouse model of Usher syndrome type 1c. Nat Biotechnol, 35, 264–272. 28165476 10.1038/nbt.3801 PMC5340578

[R34] Pan X. Huang P. Ali S. S. Renslo B. Hutchinson T. E. Erwin N. Greenberg Z. Ding Z. Li Y. Warnecke A. Fernandez N. E. Staecker H. He M. (2023). CRISPR-Cas9 engineered extracellular vesicles for the treatment of dominant progressive hearing loss. bioRxiv doi:10.1101/2023.09.14.557853. 10.1126/scitranslmed.adn3993 PMC13051476 41223249

[R35] Peters C. W. Hanlon K. S. Ivanchenko M. V. Zinn E. Linarte E. F. Li Y. Levy J. M. Liu D. R. Kleinstiver B. P. Indzhykulian A. A. Corey D. P. (2023). Rescue of hearing by adenine base editing in a humanized mouse model of Usher syndrome type 1F. Mol Ther, 31, 2439–2453. 37312453 10.1016/j.ymthe.2023.06.007 PMC10421997

[R36] Pietola L. Aarnisalo A. A. Abdel-Rahman A. Västinsalo H. Isosomppi J. Löppönen H. Kentala E. Johansson R. Valtonen H. Vasama J. P. Sankila E. M. Jero J. (2012). Speech recognition and communication outcomes with cochlear implantation in Usher syndrome type 3. Otol Neurotol, 33, 38–41. 22143301 10.1097/MAO.0b013e31823dbc56

[R37] Remjasz-Jurek A. Clarós P. Clarós-Pujol A. Pujol C. Clarós A. (2023). Outcomes of cochlear implantation in children with Usher syndrome: A long-term observation. Eur. Arch. Otorhinolaryngol, 280, 2119–2132. 36242610 10.1007/s00405-022-07670-7

[R38] Sahoo L. Sahoo K. S. Nayak N. K. Behera A. (2022). Outcomes of hearing aid and cochlear implantation in case of congenital non-syndromic bilateral severe to profound sensorineural hearing loss: An observational study. Indian J Otolaryngol Head Neck Surg, 74(Suppl 1, 200–206. 36032847 10.1007/s12070-020-01967-x PMC9411362

[R39] Schott J. W. Huang P. Morgan M. Nelson-Brantley J. Koehler A. Renslo B. Büning H. Warnecke A. Schambach A. Staecker H. (2023). Third-generation lentiviral gene therapy rescues function in a mouse model of Usher 1B. Mol Ther, 31, 3502–3519. 37915173 10.1016/j.ymthe.2023.10.018 PMC10727968

[R40] Tropitzsch A. Schade-Mann T. Gamerdinger P. Dofek S. Schulte B. Schulze M. Fehr S. Biskup S. Haack T. B. Stöbe P. Heyd A. Harre J. Lesinski-Schiedat A. Büchner A. Lenarz T. Warnecke A. Müller M. Vona B. Dahlhoff E. Holderried M. (2023). Variability in cochlear implantation outcomes in a large German cohort with a genetic etiology of hearing loss. Ear Hear, 44, 1464–1484. 37438890 10.1097/AUD.0000000000001386 PMC10583923

[R41] Velde H. M. Reurink J. Held S. Li C. H. Z. Yzer S. Oostrik J. Weeda J. Haer-Wigman L. Yntema H. G. Roosing S. Pauleikhoff L. Lange C. Whelan L. Dockery A. Zhu J. Keegan D. J. Farrar G. J. Kremer H. Lanting C. P. Pennings R. J. E. (2022). Usher syndrome type IV: Clinically and molecularly confirmed by novel ARSG variants. Hum Genet, 141, 1723–1738. 35226187 10.1007/s00439-022-02441-0 PMC9556359

[R42] Walls W. D. Azaiez H. Smith R. J. H. (2025). Hereditary Hearing Loss Homepage: Usher Syndrome. Retrieved May 1, 2025, from https://hereditaryhearingloss.org/usher

[R43] Wu C. C. (2024). Application of genetic information to cochlear implantation in clinical practice. J Audiol Otol, 28, 93–99. 38695054 10.7874/jao.2024.00080 PMC11065544

