# Supplementary material for: Multicenter Natural History Study and Long-Term Cochlear Implant Outcomes in Usher Syndrome Subtypes
Source: Ear Hear. 2026 Apr 23;47(5):1288–97. doi: 10.1097/AUD.0000000000001822 (PMC13465691; doi:10.1097/AUD.0000000000001822)
Supplement: Supplementary file 1 [file aud-47-1288-s001.pdf]

Multicenter natural history study and long-term cochlear implant outcomes in Usher syndrome subtypes

Supplemental Digital Content

Subtype	Gene	NM Number	cDNA Variant	Protein Variant	Type of Mutation	Classification	N	%
USH1B	MYO7A	NM_000260.4	c.2005C>T	p.Arg669Ter	Nonsense	Pathogenic/Likely pathogenic	1	2.2
USH1B	MYO7A	NM_000260.4	c.3892G>A	p.Gly1298Arg	Missense	Pathogenic/Likely pathogenic	1	2.2
USH1B	MYO7A	NM_000260.4	c.397del	p.His133Thrfs	Frameshift	Pathogenic	1	2.2
USH1B	MYO7A	NM_000260.4	c.5753T>G	p.Val1918Gly	Missense	Uncertain significance	1	2.2
USH1B	MYO7A	NM_000260.4	c.2542C>T	p.Arg848Trp	Missense	Uncertain significance	1	2.2
USH1B	MYO7A	NM_000260.4	c.4715T>C	p.Leu1572Pro	Missense	Likely pathogenic	1	2.2
USH1B	MYO7A	NM_000260.4	c.4838del	p.Asp1613Valfs*32	Frameshift	Pathogenic	2	4.3
USH1B	MYO7A	NM_000260.4	c.1369G>A	p.Ala457Thr	Missense	Uncertain significance	1	2.2
USH1B	MYO7A	NM_000260.4	c.1667G>T	p.Gly556Val	Missense	Likely pathogenic	1	2.2
USH1F	PCDH15	NM_001384140.1	c.131T>C	p.Val44Ala	Missense	Uncertain significance	2	4.3
USH1F	PCDH15	NM_001384140.1	c.2422A>T	p.Lys808Ter	Nonsense	None	2	4.3
USH2A	USH2A	NM_206933.4	c.653T>A	p.Val218Glu	Missense	Pathogenic	3	6.5
USH2A	USH2A	NM_206933.4	c.13576C>T	p.Arg4526Ter	Nonsense	Pathogenic	1	2.2
USH2A	USH2A	NM_206933.4	c.14413G>A	p.Val4805Ile	Missense	Uncertain significance	1	2.2
USH2A	USH2A	NM_206933.4	c.12067-2A>G		Splice acceptor	Pathogenic	2	4.3
USH2A	USH2A	NM_206933.4	c.949C>A	p.Arg317=	Synonymous	Pathogenic	1	2.2
USH2A	USH2A	NM_206933.4	c.2299delG	p.Glu767Serfs*21	Frameshift	Pathogenic	2	4.3
USH2A	USH2A	NM_206933.4	c.802G>A	p.Gly268Arg	Missense	Pathogenic/Likely pathogenic	1	2.2
USH2A	USH2A	NM_206933.4	c.6782G>A	p.Trp2261Ter	Nonsense	Pathogenic	1	2.2
USH2A	USH2A	NM_206933.4	c.11864G>A	p.Trp3955*	Nonsense	Pathogenic	2	4.3

USH2A	USH2A	NM_206933.4	c.920_923dup	p.His308fs	Frameshift	Pathogenic	1	2.2
USH2A	USH2A	NM_206933.4	c.293_294delinsGCAG	p.Thr98Serfs*48	Frameshift	Uncertain significance	1	2.2
USH2A	USH2A	NM_206933.4	c.9424G>T	p.Gly3142Ter	Nonsense	Pathogenic/Likely pathogenic	1	2.2
USH2A	USH2A	NM_206933.4	c.8681+3960A>G		Splice acceptor	Likely pathogenic	1	2.2
USH2A	USH2A	NM_206933.4	c.7524del	p.Arg2509Glyfs*19	Frameshift	Pathogenic	1	2.2
USH2A	USH2A	NM_206933.4	c.7706_7707delAT	p.Tyr2569Serfs	Frameshift	None	1	2.2
USH2A	USH2A	NM_206933.4	c.1876C>T	p.Arg626Ter	Nonsense	Pathogenic	2	4.3
USH2A	USH2A	NM_206933.4	c.10544A>G	p.Asp3515Gly	Missense	Uncertain significance	1	2.2
USH2A	USH2A	NM_206933.4	c.8559-2A>G		Splice acceptor	Pathogenic	2	4.3
USH2A	USH2A	NM_206933.4	c.6806-2A>C		Splice acceptor	Uncertain significance	1	2.2
USH2A	USH2A	NM_206933.4	c.8396del	p.Asn2799Valfs*31	Frameshift	Uncertain significance	2	4.3
USH2A	USH2A	NM_206933.4	c.14402_14403del	p.Tyr4801fs	Frameshift	Pathogenic/Likely pathogenic	1	2.2
USH2A	USH2A	NM_206933.4	c.1991G>T	p.Cys664Phe	Missense	Likely pathogenic	1	2.2
USH2C	ADGRV1	NM_032119.4	c.7129C>T	p.Arg2377Ter	Nonsense	Pathogenic/Likely pathogenic	1	2.2
USH2C	ADGRV1	NM_032119.4	c.15157G>T	p.Glu5053Ter	Nonsense	None	1	2.2

Supplementary Table 1: USH mutations and their consequences.

Note that heterozygous patients have two different mutations, therefore there can be more mutations than patients for a given subtype.

Abbreviations: NM … NCBI Reference Sequence mRNA
